# Semi-Empirical Prediction of Residual Stress Profiles in Machining IN718 Alloy Using Bimodal Gaussian Curve

**DOI:** 10.3390/ma12233864

**Published:** 2019-11-22

**Authors:** Penghao Dong, Huachen Peng, Xianqiang Cheng, Yan Xing, Wencheng Tang, Xin Zhou

**Affiliations:** 1 School of Mechanical Engineering, Jiulong Lake Campus, Southeast University, Nanjing 211189, China; dongpenghao@outlook.com (P.D.); jsszphuach1992@163.com (H.P.); chengxq_1994@163.com (X.C.); 101000185@seu.edu.cn (W.T.); 2 Shenyang Liming Aero-Engine (Group) Ltd., Shenyang 110000, China; zhou525157569xin@126.com

**Keywords:** semi-empirical prediction, residual stresses, bimodal Gaussian fit, finite element method, Inconel 718

## Abstract

Residual stresses are often imposed on the end-product due to mechanical and thermal loading during the machining process, influencing the distortion and fatigue life. This paper proposed an original semi-empirical method to predict the residual stress distribution along the depth direction. In the statistical model of the method, the bimodal Gaussian function was innovatively used to fit Inconel 718 alloy residual stress profiles obtained from the finite element model, achieving a great fit precision from 89.0% to 99.6%. The coefficients of the bimodal Gaussian function were regressed with cutting parameters by the random forest algorithm. The regression precision was controlled between 80% and 85% to prevent overfitting. Experiments, compromising cylindrical turning and residual stress measurements, were conducted to modify the finite element results. The finite element results were convincing after the experiment modification, ensuring the rationality of the statistical model. It turns out that predicted residual stresses are consistent with simulations and predicted data points are within the range of error bars. The max error of predicted surface residual stress (SRS) is 113.156 MPa, while the min error is 23.047 MPa. As for the maximum compressive residual stress (MCRS), the max error is 93.025 MPa, and the min error is 22.233 MPa. Considering the large residual stress value of Inconel 718, the predicted error is acceptable. According to the semi-empirical model, the influence of cutting parameters on the residual stress distribution was investigated. It shows that the cutting speed influences circumferential and axial MCRS, circumferential and axial depth of settling significantly, and thus has the most considerable influence on the residual stress distribution. Meanwhile, the depth of cut has the least impact because it only affects axial MCRS and axial depth of settling significantly.

## 1. Introduction

Nickel alloys represent a significant metal portion of aircraft structural and engine components [1]. Among nickel alloys, Inconel 718 is used most extensively due to the excellent properties, including fatigue resistance, oxidation resistance and corrosion resistance. The surface integrity of machined Inconel 718 is always in a significant concern, and in the indicators of the surface integrity, the residual stress of the surface layer is in great importance. The residual stress is often imposed on the end-product during the machining process, influencing distortion and fatigue life. The large machining deformations are often observed due to the machining-induced residual stresses [2]. Hence, it is essential to comprehend the mechanism of the residual stress generation and develop the prediction method of the residual stress.

Extensive researches have been carried out to obtain the mechanism of the residual stress generation. Residual stresses are generally affected by two factors: (1) plastic deformation and (2) changes in the volume of materials associated with thermal gradients and metallurgical alterations in structure [3]. Plastic deformations are linked to the thermo-mechanical loadings during machining. It is known that plastic deformations and thermo-mechanical loadings are caused and/or supported by many parameters such as cutting parameters, tool parameters and workpiece materials [1]. Consequently, these parameters influence residual stresses indirectly. Besides, residual stresses are not only relevant to macro factors such as thermo-mechanical loadings, but also relevant to the microstructure of materials. According to one paper [4], cutting parameters such as depth of cutting, feed rate and cutting speed are linked to the microstructural detail of materials. Thus, cutting parameters are linked to residual stresses both in macro and micro aspects. Residual stresses are often divided into tensile residual stresses and compressive residual stresses. It is generally observed that by increasing cutting speed, tensile residual stresses tend to become more compressive, while increases in depth of cut and feed rate have some effect on making the residual stresses more tensile at the surface and more compressive in the peak compressive depth [5]. As for the effect of tool parameters, in the turning process, nose radius and entrance angle influence residual stresses, while the rake angle plays a minor role [6]. Besides, the mechanical properties of machined material profoundly influence the level of residual stresses [7]. 

A great many prediction models of residual stresses have been developed based on the mechanism of the residual stress generation, including analytical models, finite element method (FEM) models and empirical models. A research paper developed an enhanced analytical model for residual stress prediction [8], taking into account the effect of the flank face wear length. The established 2-D modeling of Ti-6Al-4V in this paper demonstrated that the value tensile residual stress increases and the depth of residual stress distribution is more profound as the machined surface temperature increases. Kun Huang and Wenyu Yang [9] proposed an analytical model of residual stress formation. A conclusion was made that different initial stress values of one point would lead to different residual stress values, and thus, the initial stress was considered in this model. Similarly, Omar Fergani [10] also proposed an analytical algorithm to predict residual stresses in multi-step machining, including a regeneration algorithm considering the initial stress left by the previous machining step. 

The finite element method is a powerful method for modeling the residual stress prediction. T. Özel [11] used 3D finite element simulation to predict machining induced residual stress in Ti-6Al-4V and IN100 alloys, and the predicted stress field was compared against measured residual stresses. Ma [12] developed a FEM analysis model to study the evolution process of residual stress field during successive machining, claiming that the influence of machining process on the in-depth stress distribution also depended on the stress before this machining pass. A research paper of tool wear [13] also developed a FEM model to predict the residual stress considering the effect of tool wear. The results of the finite element model for the sharp tool were in high agreement with experimental results, while for the tool with a wear land, the results had a deviation from the validation tests in the compression zone. Thus, further research is needed to conduct to eliminate the sources of error in the finite element numerical model. One paper [14] developed a hybrid modeling approach by ABAQUS/Explicit and user-defined subroutine to overcome excessive element distortion problem at a small length scale in FEM. Undoubtedly, the built model could be used to simulated cutting forces, chip morphology and residual stresses under large-deformation circumstances.

The empirical modeling method has also been conducted to predict the residual stress field. Empirical models often utilize statistical methods, and they are valid for the ranges of the experiments conducted [15]. Some researchers used polynomial fits [16,17] for fitting the experimental residual stress profiles to develop empirical models. Based on previous researches, D. Ulutan [18] proposed an empirical model for residual stress profile in machining nickel-based superalloys. The paper used sinusoidal decay function to fit the residual stress profile and particle swarm optimization to optimize the error between the experimental and model data. Liang Tan [19] perfected the method from D. Ulutan by studying the evolution of the residual stress after milling, polishing and shot peening. Besides, Junteng Wang [20] also used a similar method from D. Ulutan and Liang Tan to predict the residual stress, and furthermore to predict the distortion induced by the residual stress.

Overall, the analytical modeling method has been developed well in 2D modeling, while it still has limitations in 3D modeling. The finite element method can really solve machining modeling problem, but it can be still improved in the aspect of time-consuming. The empirical modeling method can get the most accurate residual stress data but with great economic and time costs. There are still little papers that consider to combine two modeling methods and utilize both advantages.

**Figure 1:**
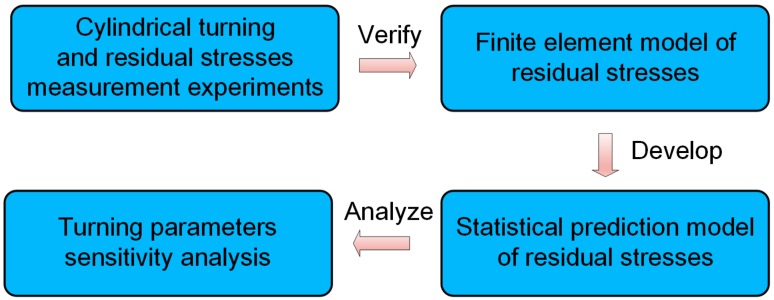
The synoptic realization steps of the proposed residual stresses prediction method.

In this paper, a semi-empirical model has been developed, consisting of the finite element modeling and statistical modeling. Thus, the model can be highly efficient as the empirical model, and meanwhile, avoid too much time-consuming on computation and many experimental works. The research procedures are shown in Figure 1. Turning process and residual stress measurement experiments were firstly conducted. Then a FEM model of residual stresses was developed and verified by the experimental data. Afterwards, a statistical prediction model of residual stresses was established from the FEM data, comprising the innovative combination of bimodal Gaussian fitting and random forest regression. The sensitivity analysis of turning parameters, aiming to investigate the impact of cutting parameters on the residual stress distribution, was also presented.

## 2. Methods

### 2.1. FEM Modelling

In order to obtain adequate residual stress profiles for later statistical prediction, a FEM model is desirable. Considering both accuracy and efficiency of simulations, the complexity of the model is also required to be balanced. Therefore, the simplified 3D cutting model was selected. The finite element simulation software is AdvantEdge (V7.4015, Third Wave Systems, Minneapolis, MN, USA).

**Figure 2:**
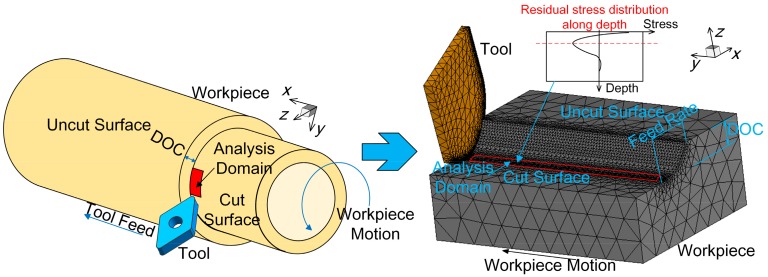
The modeling simplification of the cylindrical turning.

As shown in Figure 2, the workpiece is a tube part, whose small portion was selected as the analysis domain for reducing the computation time. Therefore, the turning model could be converted to the simplified 3D model. After the turning process, a cooling process was performed to obtain the final residual stress profiles.

Turning parameters, including feed rate (x direction), depth of cut (z direction) and cutting speed (y direction), were set in the cutting model. DOC is the abbreviation of the depth of cut. Considering that the analysis domain is minimal, the curvature of the workpiece is neglected as shown. 

The cutting tool was produced by Sandvik company (Stockholm, Sweden), and the finite element geometry model and position of the cutting tool were generated using parameters from the Sandvik website. AdvantEdge could automatically generate the cutting tool when all the tool parameters were put in. The coating material of the tool is TiAlN. The material of the tool is Carbide-Grade-M. The tool has a 0.02 mm edge radius and a 1.2 mm nose radius, with 6° clearance angle, −6° rake angle, −17.5° lead angle and −7° inclination angle. The properties of the cutting tool were input, and then the tetrahedral mesh was automatically generated, with 0.3 mm maximum element size and 0.015 mm minimum element size.

The workpiece was modeled as an elastic-plastic body, and residual stresses would be influenced both by elastic and plastic strain. In the elastic strain region, Young’s modulus and Poisson’s ratio represent mechanical properties of materials and are utilized to compute stress until the yield stress is reached and plastic deformation begins [21]. The workpiece material is Inconel 718. Young’s modulus of Inconel 718 is 204 GPa, and Poisson’s ratio is 0.3. In the plastic strain region, the influence of strain, strain rate and temperature on flow stress should be considered, which is essential in finite element analysis. Thus, a variety of constitutive models have been proposed to describe the metal cutting process, some of which were improved to more accurately depict the plastic strain of unique materials [22]. The power law constitutive model, as represented in Equation (1), was used in this paper.

(1) $$\sigma (\varepsilon^{p},\dot{\varepsilon })=g(\varepsilon^{p})\times \Gamma (\dot{\varepsilon })\times \Theta (T),$$

where $g(\varepsilon^{p})$ is strain hardening, $\Gamma (\dot{\varepsilon })$ is strain rate sensitivity and Θ(T) is thermal softening. Jiang [23] elaborated the details of this equation, compromising the specific equation of $g(\varepsilon^{p})$, $\Gamma (\dot{\varepsilon })$ and Θ(T) as followed. 

The strain hardening $g(\varepsilon^{p})$ is defined by Equations (2) and (3):

(2) $$g(\varepsilon^{p})=\sigma_{0}{\left(1+\frac{\varepsilon^{p}}{\varepsilon_{0}^{p}}\right)}^{1/n},\;\mathrm{if}\;\varepsilon^{p}<\;\varepsilon_{cut}^{p},$$

(3) $$g(\varepsilon^{p})=\sigma_{0}{\left(1+\frac{\varepsilon_{cut}^{p}}{\varepsilon_{0}^{p}}\right)}^{1/n},\;\mathrm{if}\;\varepsilon^{p}\geq \;\varepsilon_{cut}^{p},$$

where $\sigma_{0}$ is the initial yield stress, $\varepsilon^{p}$ is the plastic strain, $\varepsilon_{0}^{p}$ is the reference plastic strain, $\varepsilon_{cut}^{p}$ is the cut off strain and 1/n is the strain hardening exponent.

The thermal softening Θ(T) is defined by Equations (4) and (5):

(4) $$\Theta (T)=c_{0}+c_{1}T^{1}+c_{2}T^{2}+c_{3}T^{3}+c_{4}T^{4}+c_{5}T^{5},\;\mathrm{if}\;T\;<\;T_{cut},$$

(5) $$\Theta (T)=\Theta (T_{cut})\left(1-\frac{T-T_{cut}}{T_{melt}-T_{cut}}\right),\;\mathrm{if}\;T\;\geq \;T_{cut},$$

where $c_{0}$ through $c_{5}$ are coefficients for the polynomial fit, T is the temperature, $T_{cut}$ is the linear cut off temperature and $T_{melt}$ is the melting temperature.

The strain rate sensitivity $\Gamma (\dot{\varepsilon })$ is defined by Equations (6) and (7):

(6) $$\Gamma (\dot{\varepsilon })={\left(1+\frac{\dot{\varepsilon }}{{\dot{\varepsilon }}_{0}}\right)}^{\frac{1}{m_{1}}},\;\mathrm{if}\;\dot{\varepsilon }\;\leq {\dot{\varepsilon }}_{t},$$

(7) $$\Gamma (\dot{\varepsilon })={\left(1+\frac{\dot{\varepsilon }}{{\dot{\varepsilon }}_{0}}\right)}^{\frac{1}{m_{2}}}{\left(1+\frac{{\dot{\varepsilon }}_{t}}{{\dot{\varepsilon }}_{0}}\right)}^{\frac{1}{m_{1}}-\frac{1}{m_{2}}},\;\mathrm{if}\;\dot{\varepsilon }\;>{\dot{\varepsilon }}_{t},$$

where $\dot{\varepsilon }$ is strain rate, ${\dot{\varepsilon }}_{0}$ is reference plastic strain rate, ${\dot{\varepsilon }}_{t}$ is strain rate where the transition between low and high strain rate sensitivity occurs, $m_{1}$ is the low strain rate sensitivity coefficient and $m_{2}$ is the high strain rate sensitivity coefficient. Default constitutive model coefficient values of Inconel 718 in the software were used in this paper.

The workpiece material is Inconel 718, with 1613 MPa ultimate tensile strength and 1103 MPa yield strength. The tetrahedral mesh was automatically generated in the workpiece, with 0.5 mm maximum element size, 0.06 mm minimum element size and 0.03 mm adaptive remeshing parameters. Elements in the cutting zone determined by adaptive remeshing parameters would be refined as the contact area between the tool and the workpiece changed.

Then process parameters were input. Afterwards, the simulation was conducted with the residual stress option selected. The cooling simulation would be conducted only when the residual stress option was selected, and in this case, the workpiece was cooled to ambient temperature, which was 20 °C. In order to get accurate simulated residual stresses, the element of the adaptive remeshing area would remain small, with 0.03 mm element size, when the cutting zone went further.

### 2.2. Bimodal Gaussian Function Fitting

The residual stress is generated by mechanical and thermal loading during the machining process. Ma, Yuan [24] illustrated the deformation mechanism of the residual stress due to thermo-mechanical loadings. Typically, the residual stress is divided into the compressive residual stress and the tensile residual stress. 

**Figure 3:**
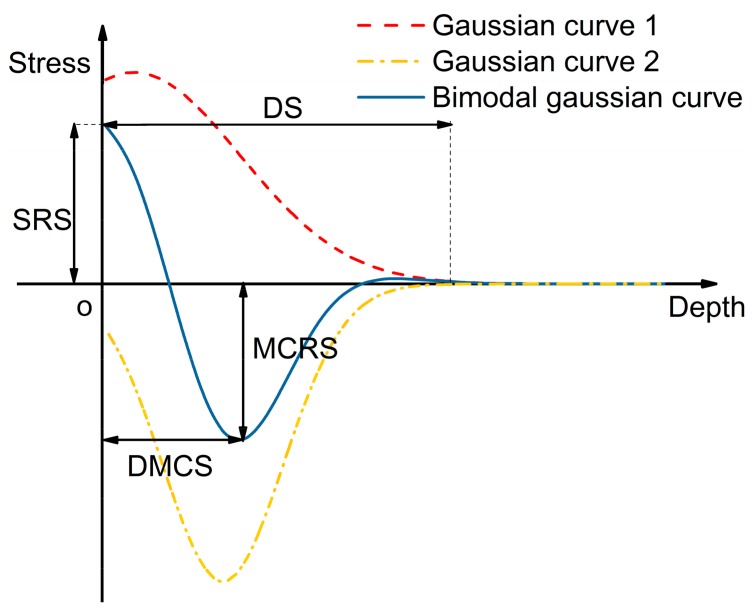
The bimodal Gaussian curve.

As shown in Figure 3, under the effect of plastic and elastic strain of the material arising from the thermal-mechanical loadings, the residual stress distribution along the depth presents a similar hook-shaped distribution curve. There are four indicators to the distribution curve, which are the surface residual stress (SRS), the maximum compressive residual stress (MCRS), the depth of the maximum compressive residual stress (DMCS) and the depth of settling (DS). The distribution curve can be fitted using appropriate functions.

Some investigations have been conducted on the model of fitting residual stress profiles. One polynomial fitting model has been proposed [16], and it can fit the residual stress well due to the flexibility of the polynomial fit. Based on specific residual stress profiles, the polynomial can be set to specific orders. The number of coefficients of a polynomial function can be adjusted. In order to achieve high fitting accuracy, orders of the polynomial have to be determined when fitting new residual stress profiles. Besides, one sinusoidal decay fitting model has also been proposed [18], and this model is concise, having only four function coefficients. The sinusoidal decay fitting model fits the residual stress well, and it is an oscillation model, achieving fitting accuracy (R^2^) varied from 67% to 93%. Yang [15] improved sinusoidal decay fitting the model, making the fitting accuracy (R^2^) varied from 81.7% to 99.2%.

Based on the distribution rule of the residual stress, the bimodal Gaussian function, which is the superposition of two two-dimensional Gaussian distribution functions, can also be used in fitting the residual stress profiles. The Gaussian distribution is also called the normal distribution. The function curve is shown in Figure 3. One Gaussian curve is added to another, becoming the bimodal Gaussian curve. With such a function, the number of coefficients is fixed. Thus, it cannot become complicated, and it is always concise. The function also converges to zero very fast, making the function fitting the residual stress well. Such a function can be represented using Equation (8) when the value of n is 2, having six coefficients.

(8) $$\sigma (z)=\sum_{i=1}^{n}\frac{A_{i}}{w_{i}\sqrt{\pi /2}}e^{-2\frac{{\left(z-z_{ci}\right)}^{2}}{w_{i}^{2}}},$$

where σ(z) is the value of the residual stress. $A_{i}$ is the amplitude constant. $w_{i}$ is the standard deviation. z is the depth to the machined surface. $z_{ci}$ is the expectation of the distribution.

However, six coefficients were too redundant for fitting the residual stress distribution curve. During the fitting process, the value of $z_{c1}$ in Gaussian curve 1 was all close to zero. Besides, after many attempts, the bimodal Gaussian curve was found that it still performed well in fitting when $w_{i}$ was fixed as 0.13. Thus, $w_{i}$ and $z_{c1}$ were fixed as 0.13 and 0 to make the fitting function more laconic. The Equation (8) can be expressed as Equation (9). Equation (9) was found that it could further improve the fitting accuracy, making the accuracy (R^2^) varied from 89.0% to 99.6%.

(9) $$\sigma (z)=\frac{A_{1}}{0.13\sqrt{\pi /2}}e^{-2\frac{z^{2}}{0.0169}}+\frac{A_{2}}{w_{2}\sqrt{\pi /2}}e^{-2\frac{{\left(z-z_{c2}\right)}^{2}}{w_{2}^{2}}}.$$

### 2.3. Random Forest Regression

After the fitting of residual stress profiles, a regression model needs to be established for describing the relationship between coefficients of the bimodal Gaussian function and cutting parameters. The relation of cutting parameters and the residual stress distribution is then finished, achieved the purpose of predicting residual stress profiles using cutting parameters. Typically, a regression model needs a regression function to represent the relationship between dependent variables and independent variables. Researchers need to know the general relation to determine the appropriate regression function. However, the general relation between coefficients of bimodal Gaussian fitting function and cutting parameters is complicated to evaluate. In this case, a regression method that does not need a regression function is of great importance.

**Figure 4:**
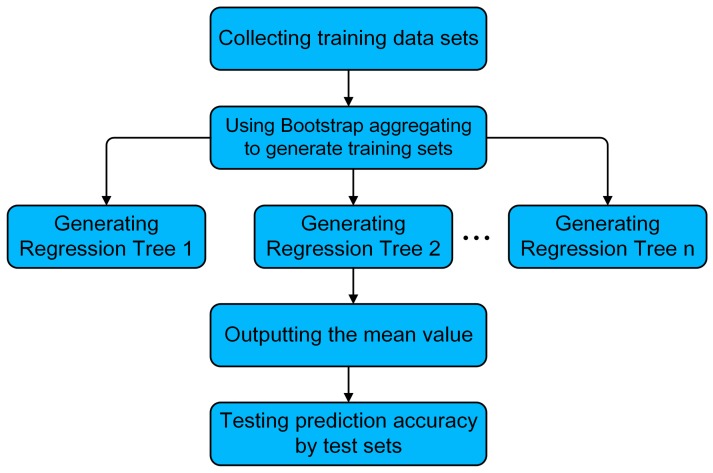
The flowchart of the random forest algorithm.

The regression can be made using the random forest regression method without a regression function expression. The random forest algorithm can operate the task by constructing a multitude of regression trees at training time and outputting the mean prediction of the individual trees [25]. As shown in Figure 4, the training sets, which are composed of cutting parameters and function coefficients (responses), are collected firstly. Then with training sets, random samples are selected repeatedly using the bagging [26] method. Samples must be put back to the training sets after one sampling. Regression trees [27] are fitted to these samples, which can be expressed by Equation (10). After training, predictions for test samples can be made by averaging the predictions from all individual regression trees, so the prediction accuracy is tested. Equation (11) is the averaging function.

(10) $$C_{i}=f_{i}(a_{p},f,v),$$

where $C_{i}$ is one coefficient of the bimodal Gaussian function. $a_{p}$ is the depth of cut. f is the feed rate. v is the cutting speed.

(11) $${\bar{C}}_{i}=\frac{1}{N}f_{i}(a_{p}^{\prime },f^{\prime },v^{\prime }),$$

where ${\bar{C}}_{i}$ is one function coefficient’s prediction value. N is the number of regression trees. $a_{p}^{\prime }$, $f^{\prime }$, $v^{\prime }$ are testing values.

## 3. Experimental Procedures

### 3.1. Workpiece and Cutting Tool

The researched material was Inconel 718 with 43 HRC hardness, and the heat treatment was solution and aging. The main chemical composition of nickel alloy Inconel 718 is shown in Table 1. The workpiece was a circular tube shape, with a 76 mm external diameter, 8.8 mm thickness and 200 mm length.

**Figure 5:**
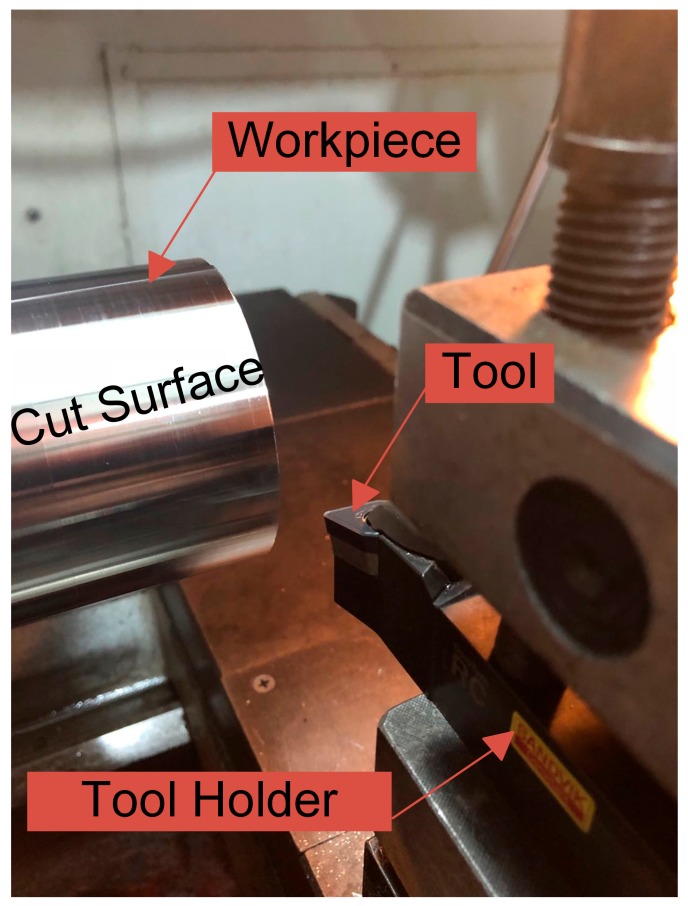
Details of the turning experiments.

The cylindrical turning tool and tool holder, shown in Figure 5, were provided by Sandvik corp. CoroPlus® ToolGuide from Sandvik was used for selecting the tool and tool holder. The tool’s model was DNMG150412-SMR1105, with PVD coating and Type D insert shape. The model of the tool holder was DDHNR 2525M 1504, with 25 mm × 25 mm connecting size. The FEM model of the cutting tool was based on parameters of the turning tool and tool holder. The turning tool mainly determined the geometry information of the FEM tool model, while the tool holder mainly determined the position information of the FEM tool model. New tools were used for each experiment to eliminate the influence of the tool wear. 

### 3.2. Cutting Parameters

All cutting experiments were conducted in the Bochi SK501 CNC lathe. Sixteen sets of cutting parameters were arranged using the Taguchi method. In order to establish the prediction model conveniently, six sets of parameters were selected from sixteen sets as experimental parameters, which are shown in Table 2. Experiment results would be used to modify the finite element model.

### 3.3. Residual Stress Measurements

**Figure 6:**
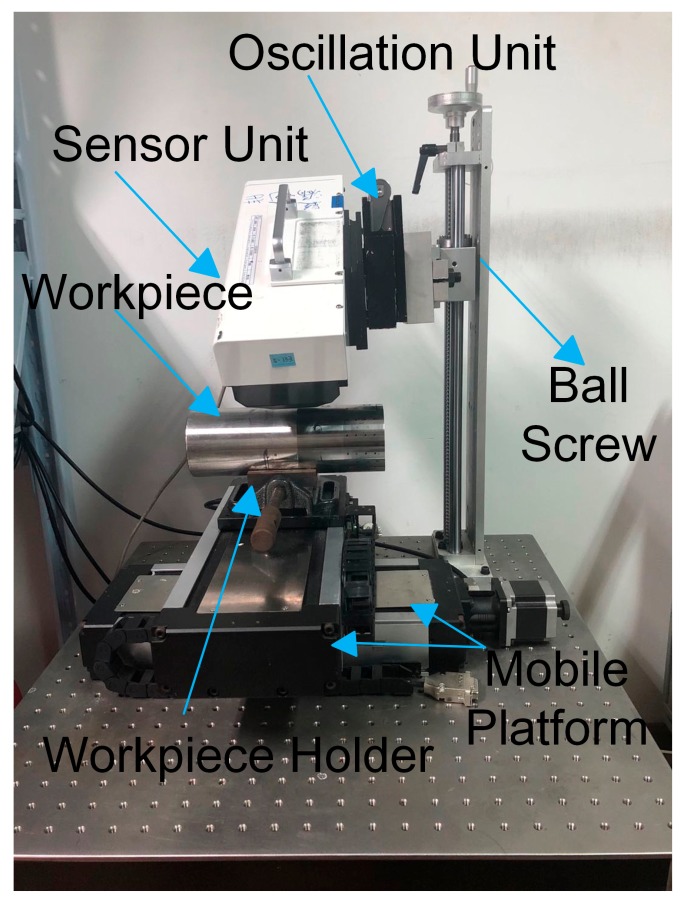
The X-ray residual stress analyzer.

**Figure 7:**
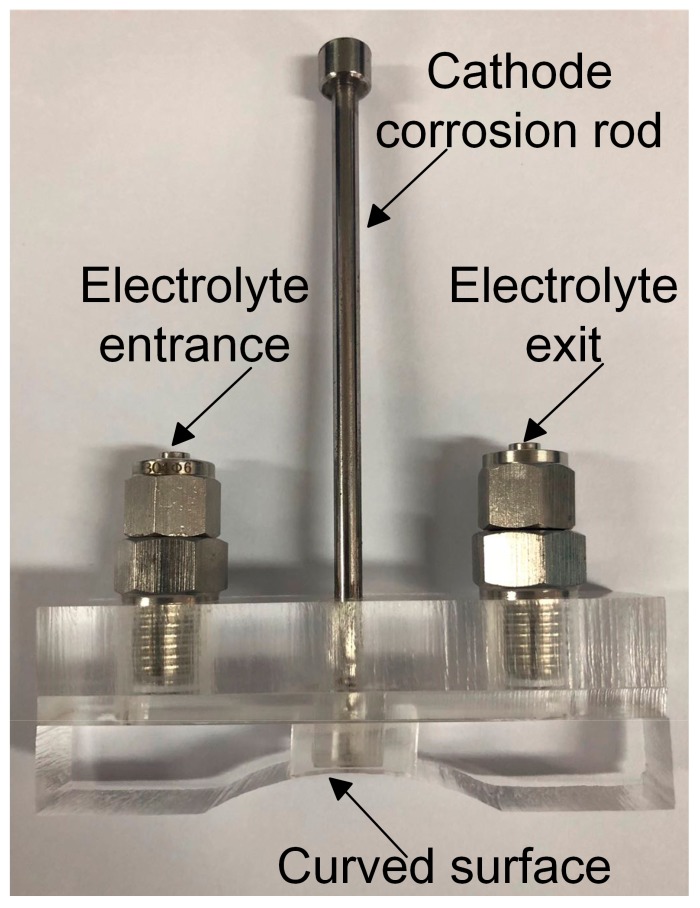
An apparatus of electrolytic corrosion.

After machining, residual stresses of all workpieces were measured by the X-ray diffraction method. As shown in Figure 6, the μ-360n X-ray residual stress analyzer was assembled by the sensor unit and the oscillation unit. The ball screw and the mobile platform were utilized to move the workpiece for measuring residual stresses of different points on the workpiece. The workpiece holder was used to fix the workpiece. Measurement parameters are listed in Table 3. Besides, in order to measure the residual stress in-depth, the electro-chemically polishing method, one of peeling methods [28], was used. Compared to chemical corrosion [29], the electro-chemically polishing method is more efficient. As shown in Figure 7, a small apparatus was designed to polish the workpiece. The cathode corrosion rod allowed corrosion of small holes of specified size on the workpiece. The electrolyte entrance and the electrolyte exit allowed the electrolyte to flow between the cathode corrosion rod and the anode workpiece. The curved surface fitted the cutting surface of the workpiece to prevent the electrolyte from flowing away. Polishing parameters are listed in Table 4.

## 4. Results and Discussions

### 4.1. Residual Stress Comparison of Simulations with Experiment Results

**Figure 8:**
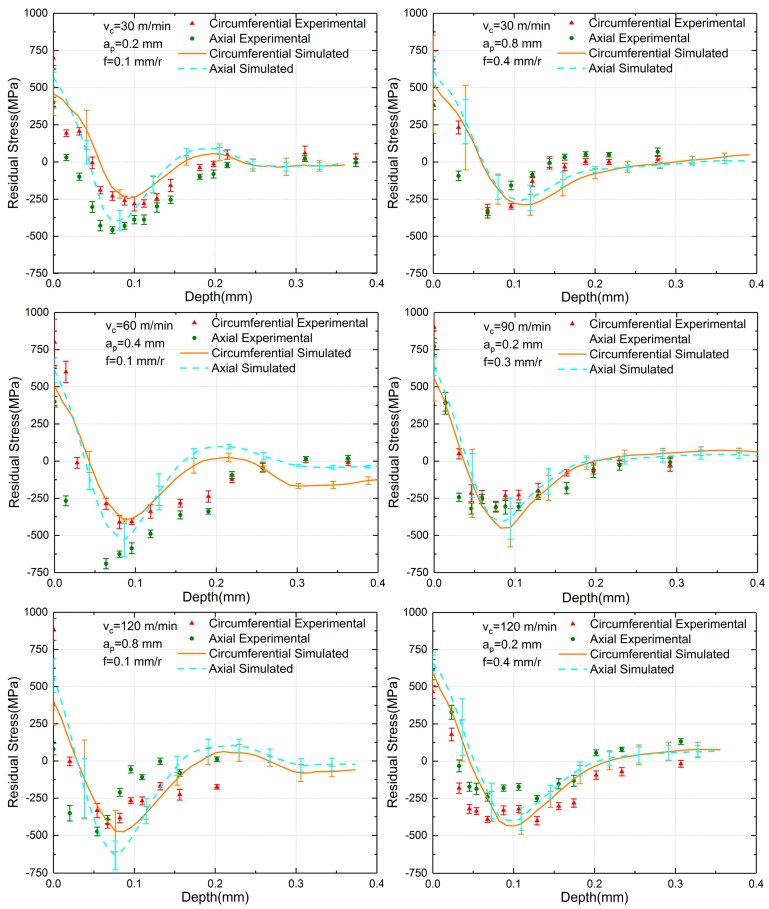
The comparation of experimental and simulated residual stress results.

A comparison between the simulated and experimental residual stresses along the depth of the cutting surface is shown in Figure 8, where the experimental residual stress is indicated in triangular and circular points at each measured depth. 

The simulated residual stress, which meets the hook-shaped distribution curve, is represented in solid lines. Standard deviations are also represented using error bars. For the six sets of parameters, it was observed that both the simulated and experimental surface residual stress was tensile, and as the depth increased, the residual stress quickly became compressive stress and then recovered to zero. As for all sets of both the simulated and experiment residual stress, the DMCS (depth of maximum compressive stress) and the DS (depth of settling) of the circumferential direction were more profound than that of the axial direction. Thus, in this aspect, the simulated results were consistent with the experimental results. When the depth of cut was also 0.2 mm and 0.4 mm, simulated results fit experimental resulted well. Meanwhile, simulated residual stresses possessed a slight deeper influence zone than experimental residual stresses when the depth of cut was 0.8 mm, but such little inaccuracy was acceptable. The SRS (surface residual stress) and MCRS (maximum compressive residual stress) of all sets also had somewhat differences with experiments. However, they were basically within the range of error bars, meaning that simulated results were reliable. Besides, simulated results were in consistency with the experimental result under varied sets of machining parameters, signifying that the simulation model was credible in other ten sets of machining parameters from the Taguchi method. 

Generally, simulated residual stresses were consistent with the experimental residual stresses, and thus, residual stress profiles obtained from the finite element simulation were reliable

### 4.2. Statistical Model of Residual Stresses

A statistical model, based on residual stress profiles from FE simulations, was established using the bimodal Gaussian curve and the random forest algorithm. Table 5 and Table 6 show the fitting function coefficient results of circumferential and axial residual stresses. The minimum of R^2^, which is the judgment criteria for regression accuracy, was 0.89, representing that the bimodal Gaussian curve could fit residual stress profiles very well.

**Figure 9:**
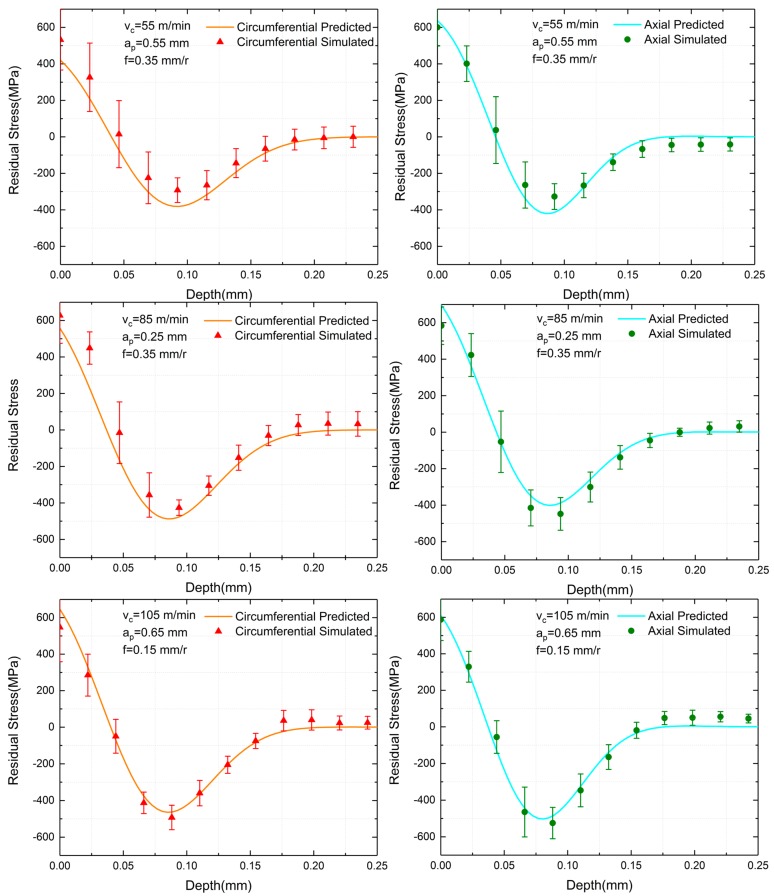
The comparation of predicted and simulated residual stress results.

In order to predict the residual stress distribution by cutting parameters, the random forest algorithm was utilized to build a regression model between cutting parameters and fitting function coefficients. All the data from Table 5 and Table 6 were used as training sets for random forest regression. In order to prevent overfitting, The R^2^ value of the random forest regression was controlled between 0.8 and 0.85. Figure 9 shows the predicted residual stress distribution and simulated residual stress profiles at three validation sets. With 55 m/min cutting speed, 0.55 mm depth of cut and 0.35 mm/r feed rate, the SRS, DMCS and DS of the prediction were in good agreement with simulation in both directions, while the predicted MCRS of two directions were somewhat higher than the simulation. As to other two validation sets, which were 85 m/min cutting speed, 0.25 mm depth of cut, 0.35 mm/r feed rate and 105 m/min cutting speed, 0.65 mm depth of cut, 0.15 mm/r feed rate, predicted curves fit simulation results perfectly in SRS, MCRS, DMCS and DS. It can be noted that all curves of the last two validation sets were within the simulated error bars. Predicted residual stress profile functions of three validation sets are shown in Table 7. Curves in Figure 9 are graphical representations of six functions, including the circumferential and axial direction. As shown in Table 7, six functions had their test numbers from one to six. Table 8 shows the max and min predicted errors of residual stress indicators. The max and min predicted errors of SRS were 113.156 MPa and 23.027 MPa, while the max and min predicted errors of MCRS were 93.025 MPa and 22.233 MPa. The residual stress value level of Inconel 718 was high, and the value often could reach 1000 MPa. Thus, predicted residual stress errors were acceptable. As for the DMCS, the max and min errors were 0.00905 mm and 0.000690 mm. DS had a 0.0142 mm max error and a 0.00149 mm min error. Depth errors were minimal.

In general, predicted residual stress distribution curves were consistent with the simulated residual stress profiles, proving that the semi-empirical prediction method proposed by this paper was convincing.

### 4.3. Sensitive Analysis

**Figure 10:**
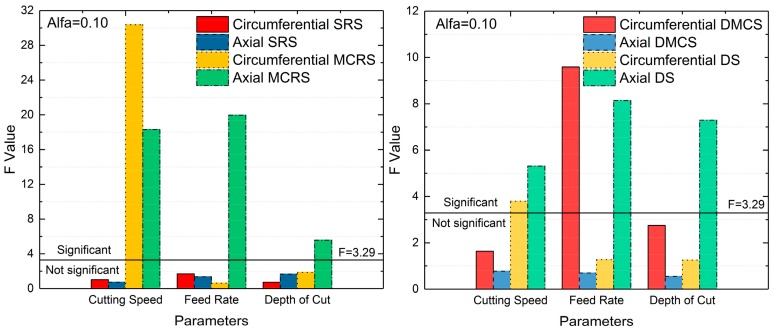
The effects of the cutting parameters on residual stress distribution indicators.

ANOVA was utilized in this paper to reveal the effects of cutting parameters on SRS, MCRS, DMCS and DS. Figure 10 shows the sensitivity analysis result. F is a statistic for examining the sensitivity, reflecting the influence degree of machining parameters on indicators, which were SRS, MCRS, DMCS and DS. The confidence interval was chosen as 0.90, and then the confidence α was 0.10. According to the F distribution table, the value of F in this sample was 3.29. When the F value of one factor exceeded 3.29, it meant the factor influenced the indicator significantly. Otherwise, the factor had little effect on the indicator. 

As can be seen in Figure 10, all three machining parameters imposed little effects on both circumferential and axial SRS. As for MCRS, the cutting speed had a significant impact on both circumferential and axial MCRS, and the feed rate and depth of cut influence the axial MCRS a lot. Besides, the feed rate affected circumferential DMCS to a great extent, while no parameters influenced axial DMCS. As to the DS, the circumferential DS was only sensitive to the cutting speed, while the axial DS was sensitive to all three parameters. 

To sum up, the cutting speed affected four indicators greatly, therefore having the most significant impact on the residual stress distribution. Feed rate influenced three indicators, also affecting the residual stress greatly. The depth of cut had the least effect on the residual stress distribution, only significantly affecting two indicators.

## 5. Conclusions

In this paper, residual stresses after cylindrical turning were investigated, and a new semi-empirical prediction method for machining residual stresses was developed using the bimodal Gaussian curve and the random forest algorithm. The impact of cutting parameters on the residual stress distribution was also investigated. The following conclusions could be made according to the investigation:

1. The finite element model built in this paper was a reliable tool to reflect the experimental turning process. Simulated residual stress distributions were compared to experimental results under six sets of machining parameters. It turned out residual stresses could be relatively accurately obtained by the simulation model, and thus simulated results could be used as the training data for the later statistical prediction model.
2. High consistency between verified simulated residual stress distributions and statistical predicted residual stress distributions was exhibited in this paper. The bimodal Gaussian curve was used in the statistical model to fit the simulated results, achieving the fitting accuracy from 89.0% to 99.6%. The random forest algorithm was utilized to build a regression model between machining parameters and fitting coefficients, and the regression accuracy was controlled between 80% and 85% to prevent the overfitting. Three validation sets were showed both in circumferential and axial directions, and it turned out predicted residual stress distribution curves were consistent with the simulated residual stress profiles in both directions. Max errors of the surface residual stress (SRS), the maximum compressive residual stress (MCRS), the depth of the maximum compressive residual stress (DMCS) and the depth of settling (DS) were 113.156 MPa, 93.025 MPa, 0.00905 mm and 0.0142 mm, which was acceptable.
3. Indicators, compromising the surface residual stress (SRS), the maximum compressive residual stress (MCRS), the depth of the maximum compressive residual stress (DMCS) and the depth of settling (DS), were investigated in the sensitivity to machining parameters. It showed that the cutting speed had the most considerable influence on these indicators, and feed rate also influenced indicators much. However, the depth of cut had the least impact on indicators.

## Figures and Tables

**Table 1:** The chemical composition of Inconel 718.

Elements	Ni	Cr	Fe	Nb	Mo	Ti
Weight%	52.860	19.085	19.15	5.085	3.105	0.710

**Table 2:** Experimental turning parameters.

Number	Feed Rate f mm/r	Depth of Cut a_p_ mm	Cutting Speed v m/min
1	0.1	0.2	30
2	0.4	0.8	30
3	0.1	0.4	60
4	0.3	0.2	90
5	0.1	0.8	120
6	0.4	0.2	120

**Table 3:** X-ray residual stress measurement parameters.

Parameters	Values
X-ray tube voltage	30.00 KV
X-ray tube current	1.20 mA
X-ray wavelength (K-Beta)	2.08480[A](Cr)
Diffraction angle (2Theta)	150.876 deg
Diffraction lattice angle (2Eta)	29.124 deg

**Table 4:** Electrolytic corrosion parameters.

Electrolytic Parameters	Values
Electrolyte	10% NaCl
Electrolyte speed	800 mL/min
Voltage	24 V
Electric current	3 A
Polishing rate	0.005 mm/s

**Table 5:** Circumferential residual stresses fitting function coefficients.

No.	Feed Rate f mm/r	Depth of Cut a_p_ mm	Cutting Speed v m/min	$A_{1}$	$A_{2}$	$w_{2}$	$z_{c2}$	R^2^
1	0.1	0.2	30	76.949	−36.866	0.0847	0.0673	0.978
2	0.2	0.4	30	79.562	−41.205	0.0788	0.0677	0.910
3	0.3	0.6	30	108.799	−70.394	0.0730	0.0864	0.985
4	0.4	0.8	30	113.572	−73.099	0.0851	0.1170	0.989
5	0.1	0.4	60	106.241	−74.913	0.0749	0.0872	0.996
6	0.2	0.2	60	120.262	−81.490	0.0656	0.0859	0.954
7	0.3	0.8	60	143.626	−88.654	0.0715	0.0919	0.986
8	0.4	0.6	60	76.651	−58.728	0.0738	0.1031	0.986
9	0.1	0.6	90	226.585	−172.272	0.0479	0.1046	0.915
10	0.2	0.8	90	85.867	−73.056	0.0767	0.0869	0.976
11	0.3	0.2	90	159.128	−116.290	0.0628	0.0975	0.960
12	0.4	0.4	90	108.665	−77.353	0.0851	0.0892	0.964
13	0.1	0.8	120	113.242	−98.431	0.0665	0.0942	0.950
14	0.2	0.6	120	155.171	−116.350	0.0665	0.0890	0.908
15	0.3	0.4	120	110.125	−75.271	0.0792	0.0783	0.890
16	0.4	0.2	120	140.745	−103.309	0.0752	0.1057	0.972

**Table 6:** Axial residual stresses fitting function coefficients.

No.	Feed Rate f mm/r	Depth of Cut a_p_ mm	Cutting Speed v m/min	$A_{1}$	$A_{2}$	$w_{2}$	$z_{c2}$	R^2^
1	0.1	0.2	30	162.795	−88.504	0.0752	0.0786	0.986
2	0.2	0.4	30	63.657	−45.976	0.0783	0.0732	0.994
3	0.3	0.6	30	129.306	−82.682	0.0705	0.0779	0.993
4	0.4	0.8	30	127.230	−69.057	0.0802	0.1054	0.993
5	0.1	0.4	60	127.431	−90.047	0.0705	0.0775	0.964
6	0.2	0.2	60	160.150	−105.493	0.0609	0.0889	0.991
7	0.3	0.8	60	108.326	−66.932	0.0774	0.0735	0.989
8	0.4	0.6	60	115.207	−58.872	0.0827	0.0772	0.987
9	0.1	0.6	90	141.248	−98.805	0.0619	0.0772	0.927
10	0.2	0.8	90	114.557	−79.764	0.0707	0.0802	0.990
11	0.3	0.2	90	159.798	−103.773	0.0637	0.0902	0.984
12	0.4	0.4	90	129.134	−84.358	0.0824	0.0925	0.986
13	0.1	0.8	120	146.977	−116.256	0.0634	0.0818	0.968
14	0.2	0.6	120	160.463	−113.094	0.0655	0.0851	0.966
15	0.3	0.4	120	116.918	−83.780	0.0757	0.0751	0.975
16	0.4	0.2	120	136.899	−85.867	0.0789	0.0938	0.979

**Table 7:** Predicted residual stress profile functions.

$\sigma (z)=\frac{A_{1}}{0.13\sqrt{\pi /2}}e^{-2\frac{z^{2}}{0.0169}}+\frac{A_{2}}{w_{2}\sqrt{\pi /2}}e^{-2\frac{{\left(z-z_{c2}\right)}^{2}}{w_{2}^{2}}}$								
Feed Rate f mm/r	Depth of Cut a_p_ mm	Cutting speed v m/min	Test No.	Direction	$A_{1}$	$A_{2}$	$w_{2}$	$z_{c2}$
0.35	0.55	55	1	Circumferential	99.691	−77.193	0.0943	0.0738
			2	Axial	123.882	−74.734	0.0769	0.0738
0.35	0.25	85	3	Circumferential	146.828	−110.668	0.0939	0.0667
			4	Axial	159.869	−98.236	0.0872	0.0661
0.15	0.65	105	5	Circumferential	155.582	−105.887	0.0894	0.0669
			6	Axial	135.573	−93.241	0.0791	0.0673

**Table 8:** Max and min predicted errors of residual stress indicators.

Indicators	Max Error	Test No.	Min Error	Test No.
SRS (MPa)	113.156	4	23.047	6
MCRS (MPa)	93.025	2	22.233	6
DMCS (mm)	0.00905	3	0.000690	1
DS (mm)	0.0142	3	0.00149	1
